# Premature Ovarian Insufficiency: Past, Present, and Future

**DOI:** 10.3389/fcell.2021.672890

**Published:** 2021-05-10

**Authors:** Seung Joo Chon, Zobia Umair, Mee-Sup Yoon

**Affiliations:** ^1^Department of Obstetrics and Gynecology, Gachon University Gil Medical Center, College of Medicine, Gachon University, Incheon, South Korea; ^2^Department of Molecular Medicine, College of Medicine, Gachon University, Incheon, South Korea; ^3^Lee Gil Ya Cancer and Diabetes Institute, Incheon, South Korea; ^4^Department of Health Sciences and Technology, GAIHST, Gachon University, Incheon, South Korea

**Keywords:** premature ovarian insufficiency, premature ovarian failure, early menopause, ovarian aging, ovary

## Abstract

Premature ovarian insufficiency (POI) is the loss of normal ovarian function before the age of 40 years, a condition that affects approximately 1% of women under 40 years old and 0.1% of women under 30 years old. It is biochemically characterized by amenorrhea with hypoestrogenic and hypergonadotropic conditions, in some cases, causing loss of fertility. Heterogeneity of POI is registered by genetic and non-genetic causes, such as autoimmunity, environmental toxins, and chemicals. The identification of possible causative genes and selection of candidate genes for POI confirmation remain to be elucidated in cases of idiopathic POI. This review discusses the current understanding and future prospects of heterogeneous POI. We focus on the genetic basis of POI and the recent studies on non-coding RNA in POI pathogenesis as well as on animal models of POI pathogenesis, which help unravel POI mechanisms and potential targets. Despite the latest discoveries, the crosstalk among gene regulatory networks and the possible therapies targeting the same needs to explore in near future.

## Introduction

Premature ovarian insufficiency (POI) is characterized by deficient ovarian sex hormones and decreased ovarian reserve, which together lead to an accelerated reduction in ovarian function and an early onset of menopause (Wesevich et al., 2020).

Premature ovarian insufficiency is a medical condition in which ovarian follicles are depleted and cease to function normally both as reproductive organs and endocrine organs in women under 40 years old (Welt, 2008; Jankowska, 2017). It is characterized by deficient ovarian sex hormones and decreased ovarian follicles that accelerate the onset of menopause (Wesevich et al., 2020). This condition often results in subfertility or infertility, as it is associated with hypoestrogenism, which causes menstrual irregularities and pregnancy failures (Ebrahimi and Akbari Asbagh, 2011). The decrease in estrogen secretion also causes a myriad of menopausal symptoms, such as hot flashes, night sweats, and insomnia. In addition, long-term consequences of premature loss of ovarian function increase the lifetime risk of skeletal fragility and cardiovascular and neurocognitive disorders (Wesevich et al., 2020).

Delayed diagnosis of POI can be attributed to mild clinical symptoms and a relative lack of awareness. POI is mostly diagnosed after menarche; however, if it occurs before menarche, it should be distinguished from gonadal dysgenesis, in which ovaries are morphologically and histologically different (Taylor et al., 2019). Despite recent progress in the field of reproductive endocrinology, various mechanisms underlying ovarian dysfunction remain vague; and the process of regulating and maintaining ovarian follicle quality and quantity needs to be further investigated. Thus, we review the histology and possible causes of POI, as well as the recent advances in elucidating the molecular intricacies of POI, along with mouse models to study this condition.

## Clinical Aspect of POI

### Diagnosis of POI

Premature ovarian insufficiency is diagnosed when a woman presents amenorrhea before 40 years of age (Torrealday et al., 2017) with an elevated serum level of pituitary gonadotropin follicle-stimulating hormone (FSH) with low levels of estradiol (E2). Serum levels of FSH and E2 are measured on at least two separate occasions with more than 4 weeks of interval, and patients that present with continuously elevated FSH levels (greater than 25 IU/L) are diagnosed with POI (Wesevich et al., 2020). Although this condition was previously referred to as premature ovarian failure (POF), some patients are known to have residual ovarian function that seldom leads to pregnancy. Therefore, the term POI was adopted by an American consensus meeting and by the European Society of Human Reproduction and Embryology (ESHRE) consensus (Welt, 2008). POI occurs in approximately 1% of the women who have not reached 40 years of age. Study of Women’s Health Across the Nation (SWAN) reported a 1.1% prevalence of POI among women; by ethnicity, 1% of Caucasian, 1.4% of African American, 1.4% of Hispanic, 0.5% of Chinese, and 0.1% of Japanese women experienced POI, and the difference in frequency among these ethnic groups was statistically significant (Wesevich et al., 2020). The prevalence of POI was higher in countries with medium and low human development indexes (Jankowska, 2017). The frequency is roughly 4–8% in women experiencing secondary amenorrhea and 10–28% in cases of primary amenorrhea (Allshouse et al., 2015). The estimated incidence rate ratio varies with age; the ratio is 1:100 cases by the age of 40 years, 1:250 cases at the age of 35 years, 1:1000 cases by 30 years, and 1:10,000 cases during the age of 18–25 years. Epidemiological studies have shown that POI incidence also depends on ethnicity (Rebar, 2009; Rudnicka et al., 2018).

### Clinical View of POI

The clinical findings in women with POI are highly variable. These symptoms are indistinguishable from those associated with menopause. They include having difficulty conceiving, and experiencing new-onset menstrual irregularity after pregnancy or after ceasing the use of birth control pills. Amenorrhea in a healthy woman for three consecutive months requires further investigation, with POI being a probable differential (Torrealday et al., 2017). Hot flashes, dyspareunia, night sweats, dry eyes, and decreased sexual desire are other typical symptoms which resemble those experienced in a menopausal or an estrogen-deficient state. However, women experiencing primary amenorrhea may never experience symptoms of hypoestrogenism. Women with POI occasionally show signs of Turner syndrome, such as short stature, webbed neck, short fourth and fifth metacarpal bones, shield-like chest, wide carrying angle elbow, low set ears, and low hairline. Turner syndrome is the most common genetic cause of POI, with these cases being usually clinically evident before menarche (Torrealday et al., 2017).

Familial syndromes with unusual features are at times associated with POI. Such manifestations include dwarfism, hearing loss, and eyelid tumors (Torrealday et al., 2017). Clinical outcomes of certain autoimmune diseases such as thyroid and adrenal autoimmunity have also been linked to POI (Kovanci and Schutt, 2015). They include patchy loss of skin pigmentation, premature graying of hair, spot baldness, candidiasis, and nail dystrophy. These women may also have symptoms of adrenal insufficiency, such as hyperpigmentation, orthostatic hypotension, salt craving, anorexia, abdominal pain, and loss of hair in the axillary and pubic areas. Importantly, there are signs of thyroid disease, such as bulging eyes, goiter, and increased or decreased heart rate. During pelvic examination, ovaries are commonly not palpable, with evidence of atrophic vaginitis, although women with enough estrogen to maintain normal vaginal mucosa have also been observed (Torrealday et al., 2017). In some other cases, the ovaries are enlarged.

### Histology of Ovaries in POI

**FIGURE 1:**
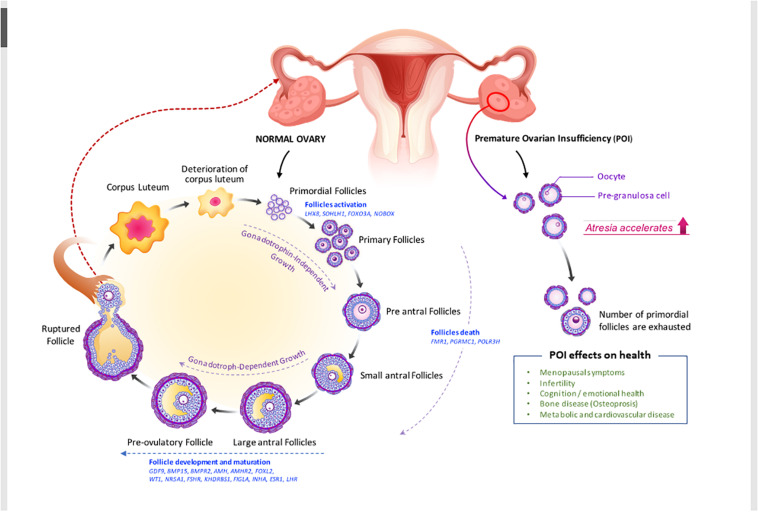
Folliculogenesis and ovulation in normal ovary versus POI ovary (impaired folliculogenesis). Under the regulation of intraovarian factors and gonadotropins, primary follicles develop into preantral and early antral follicles, which are the most susceptible to atresia, or follicle death. Then, they become preovulatory follicles, resulting in oocyte release and corpora lutea formation. Defects in folliculogenesis (e.g., decrease in primordial follicles, increase in atresia, and altered follicular maturation) causes POI. Selected genes that are involved in ovarian follicle activation, maturation, and death and the effect of POI on health (see also in Table 2 and section “Clinical view of POI”) are shown. AMHR2, anti-Müllerian hormone receptor 2; BMP15, bone morphogenic protein 15; BMPR2, bone morphogenetic protein receptor 2; FMR1, fragile X mental retardation; FSHR, follicle-stimulating hormone receptor; FOXO3A, forkhead box O3; FOXL2, forkhead box L2; GDF-9, growth differentiation factor 9; KHDRBS1, heteronuclear ribonucleoprotein particle K homology domain RNA binding S1; LHX8, LIM homeobox 8; NOBOX, newborn ovary homeobox; NR5A1, nuclear receptor subfamily 5 group A member 1; PGRMC1, progesterone receptor membrane component 1; POLR3H, RNA polymerase III subunit H; SOHLH1, spermatogenesis and oogenesis specific basic helix–loop–helix 1.

Ovarian morphology and histology in POI can be distinguished from those in gonadal dysgenesis. Ovarian follicles in gonadal dysgenesis deplete during embryogenesis or the first few years after birth; and ovaries do not have follicles but only stroma, appearing as fibrous streaks (Taylor et al., 2019). On the other hand, the ovaries of women with POI contain follicles, though these are resistant to high doses of gonadotropins (Figure 1).

Ovarian histology varies according to the phenotypes of POI. However, most antral follicles are histologically abnormal; follicles are atretic, ranging from partial sloughing to total absence of granulosa cells (Meduri et al., 2007). The detectability of serum anti-Müllerian hormone (AMH) in POI patients could be significantly correlated with the presence of 15 or more follicles in their ovaries (Meduri et al., 2007; Yoon et al., 2013; Alvaro Mercadal et al., 2015). Although the number of cases in each group was not statistically sufficient, the mean serum AMH level was 2.16 ng/ml in women with 15 or more follicles and 0.42 and 0.33 ng/ml in women without follicles and those with five or fewer follicles, respectively. Although ovarian follicles are not visible on ultrasonography, assessing serum AMH could screen POI patients who are more likely to possess follicles that can eventually grow (Yoon et al., 2013; Alvaro Mercadal et al., 2015).

Ovarian histology in POI is a definitive method of evaluating follicle conditions and ovarian reserve (Massin et al., 2004). Studying the morphological changes in this condition can help understand the extent of follicular reserve impairment and growth, and help describe the type of POI and determine the actual etiology (Massin et al., 2004). Two types of POI can be found according to histological studies: those presenting with small ovaries, deprived of follicles, and those presenting with normal-sized ovaries with partial follicular maturation. Even though atretic follicles and active primordial follicles are absent in the follicular type, the ovaries still have stroma and corpora albicantia, which consist of an eosinophilic mass surrounded by a collagen-rich connective tissue capsule. In contrast, the follicular type contains many active primordial follicles without growing follicles. Lymphoplasmacytic infiltration is sometimes observed around the primordial follicles.

Using light and transmission electron microscopy, dense connective tissue and a few corpora albicantia can be observed in the ovaries of women with POI (Zhang et al., 2019). Under light microscopy, the inner portion of the ovary, known as the medulla, and the outer cortical region are united without a clear demarcation. The distribution of fibrillar elements and cells is not homogeneous. Electron transmission microscopy shows a high concentration of fibroblasts and collagen fibers in the ovarian stroma. Furthermore, in this compartment, cells are not homogeneously distributed some regions show greater collagen deposition, while others have more cellular elements (Zhang et al., 2019). Inside corpora albicantia as well as in their periphery, fibroblasts present a high concentration of cytoplasmic myofilaments. Ultrasound scans have revealed that multiple blood vessels mixed with collagen, and few primordial follicles and active fibroblasts are distributed heterogeneously in the ovaries of women with POI (Haidar et al., 1994; Maclaran and Panay, 2011; Cox and Liu, 2014).

## Pregnancy in POI

Spontaneous pregnancies are extremely scarce in patients with POI. Women experiencing POI have menstrual irregularities that hinder their fertility. Some patients with idiopathic POI present an intermittent ovarian function and hence, their chance of conceiving spontaneously and having an uneventful pregnancy course is approximately 5% (Baek, 2018). POI is different from menopause in that ovarian insufficiency may not be permanent. Among the 25% of POI patients who can ovulate, only 5–10% can conceive (Schover, 2008; Torrealday et al., 2017). POI can be reversed depending on whether amenorrhea is primary or secondary. Primary amenorrhea is more serious than secondary amenorrhea, making reversal easier in the latter. Laboratory test results for FSH, estradiol, and inhibin B can predict the chance of POI reversal (Caroppo and D’Amato, 2012). Oocyte donation is the recommended treatment for infertility due to POI, as it has been proven to achieve a 70–80% successful pregnancy rate, although it may not be available in some countries (Torrealday et al., 2017). Immature egg cell donation is also indicated to women born with Turner syndrome, but only after undergoing cardiovascular control, as they are prone to cardiovascular mortality during pregnancy. Fertility preservation strategies, such as the cryopreservation of oocytes, ovarian tissues, and embryos, are suitable alternatives. Gonadal function can also be protected by surgically changing the position of the ovaries out of the pelvis. Ovarian cryopreservation is another option for women who have hormone-sensitive malignancies and for those who will undergo hematopoietic stem cell transplantation for aplastic anemia, sickle cell anemia, and thalassemia major. Furthermore, a new approach known as *in vitro* activation (IVA) of dominant follicles has gained relevance in the treatment of infertility (Torrealday et al., 2017). IVA disrupts the Hippo signaling pathway and stimulates phosphatidylinositol-3-kinase (PI3K) to activate dominant primordial follicles in patients with POI (Lee and Chang, 2019).

## Hormone Replacement Therapy in Premature Ovarian Insufficiency

Careful counseling of women with POI to provide emotional support is crucial since POI has been associated with physiological distress (Faubion et al., 2015). Along with this, it is important to minimize the sequelae of hypogonadism through continuous evaluation and medical treatment. Low levels of serum estrogen may cause low bone mass and be a predisposing factor for coronary heart disease. Appropriate physiological estrogen/progestin therapy is regarded as the conventional management of POI, as it ameliorates the health complications resulting from this condition, such as menopause-associated symptoms, loss of bone mineral density, fractures, and dry eye syndrome (Fenton, 2015). Hormone replacement therapy (HRT) involves the prescription of hormones to replace those that normally would be present, but are actually deficient (Fenton, 2015). HRTs include the administration of bioidentical and non-bioidentical estrogens and progestins such as norgestrel and progesterone, as well as compounding for women that require multiple hormones (Vujovic et al., 2010). Estrogen deficiency is the primary ovarian hormone deficiency in women with POI. Therefore, unless estrogen-based hormone therapy is contraindicated, estrogen treatment is required to replace the depleted estrogen. Physiological estrogen levels can be achieved with oral (micronized estradiol 1–2 mg daily or conjugated equine estrogens 0.625–1.25 mg daily) or transdermal estrogen regimens (0.1 mg daily) (Ratner and Ofri, 2001). Moreover, additive continuous or cyclic progesterone is required if women have a uterus to protect their endometrium. For cyclic therapy, a progestogen (micronized progesterone 200 mg daily or medroxyprogesterone acetate 10 mg daily for 12–14 days in a month) could be administered additively if the patient is pursuing pregnancy (Crofton et al., 2010). HRT use is not recommended in women with a history of breast and ovarian cancer, in breast-feeding mothers (as it can cause neonatal jaundice and neonatal breast enlargement), and in patients that have reached the age of 50 years, although the decision to stop HRT depends on many factors (Panay and Kalu, 2009).

Before prescribing hormone pills, POI patients should be educated to understand that their hormone therapy differs from postmenopausal hormone therapy. Many health risks increase with the use of estrogen/progestin therapy, including those of breast cancer, stroke, and cardiovascular diseases (Dragojević-Dikić et al., 2009). However, potential risks and benefits of HRTs are different in these two populations; since POI patients are generally younger than postmenopausal women, their risks of cardiovascular disease and breast cancer are significantly lower. Estrogen formulations are superior to synthetic ethinyl estradiol in terms of their benefits to bone mineral density unless a patient opts for contraception (Panay and Kalu, 2009). Estrogen-based hormone therapy should be continued up to the age of 50 years, which marks the onset of natural menopause (Panay and Kalu, 2009).

## Causes of POI (Genetic, Iatrogenic, Autoimmune, Metabolic, Infectious, or Environmental)

Premature ovarian insufficiency is usually designated as spontaneous or idiopathic POI because its etiology is mostly underdetermined (Rudnicka et al., 2018). It has been suggested that POI is prompted by underlying mechanisms such as early follicular depletion, blockage of follicular maturation or destruction of the oocyte pool, and resistant ovarian syndrome (Fraison et al., 2019). The identified causes of POI, potentially involved in those mechanisms, have been grouped into two categories: genetic and non-genetic causes. Genetic causes entail various genetic abnormalities, while non-genetic causes include autoimmune and metabolic disorders, infections, environmental factors, and iatrogenic procedures (Woad et al., 2006; Jiao et al., 2017; Rudnicka et al., 2018; Table 1).

**TABLE 1:** Causes of premature ovarian insufficiency (POI).

Genetic causes	Trisomy X (47 XXX or mosaic) (Allen et al., 2007; Chapman et al., 2015; Dawood et al., 2018; Kirshenbaum and Orvieto, 2019; Wesevich et al., 2020)
	Deletion of X chromosome (Allen et al., 2007; Chapman et al., 2015; Dawood et al., 2018; Kirshenbaum and Orvieto, 2019; Wesevich et al., 2020)
	Turner mosaic (45XO/46XX) (Allen et al., 2007; Chapman et al., 2015; Dawood et al., 2018; Kirshenbaum and Orvieto, 2019; Wesevich et al., 2020)
	Turner syndrome (Torrealday et al., 2017) Fragile X premature (Allen et al., 2007; Chapman et al., 2015; Dawood et al., 2018; Kirshenbaum and Orvieto, 2019; Wesevich et al., 2020)
	Autoimmune polyglandular syndrome (1 and 2) Blood syndrome
	BPES
	Ataxia telangiectasia
	Fanconi anemia
	Autoimmune oophoritis
	Enzyme deficiency
	• Galactosemia
	• 17^α^-hydroxylase deficiency
	• Aromatase deficiency
	Infectious diseases
	• Shigelosis
	• Chickenpox
	• Mumps oophoritis
	• Tuberculosis
	• Malaria
	• Cytomegalovirus infection

Induced/Others	Chemotherapy
	Alkylating agents
	• Cyclophosphamide, dacarbazine, chlorambucil, [-2.5pt] melphalan, busulphan, nitrogen mustard, and anthracyclines (doxorubicin)
	Substituted hydrazine (procarbazine)
	Bilateral oophorectomy
	Bilateral ovarian cystectomy
	Radiation
	Environmental toxins
	Pelvic vessel embolization

*Blepharophimosis-Epicanthus inversus syndrome*

### Genetic Aspect of POI

#### Chromosomal Abnormality and Genetic Mutations

Genetic causes of POI are highly heterogeneous and may involve interactions of various genetic defects. First-degree relatives of approximately 10–30% of idiopathic POI cases also have this condition, strongly supporting the association of POI etiology with genetics (Chapman et al., 2015; Qin et al., 2017)(Table 2). Chromosomal abnormalities, genetic polymorphisms, and single-gene mutations have been recognized as causes of POI (Wesevich et al., 2020). X-chromosomal defects linked to POI indicate that this chromosome is vital to normal ovarian function, as these defects cause POI development. X-chromosome abnormalities comprise of duplications, deletions, and translocation of the X chromosome, whereas Turner syndrome consists of total or partial deletion of one X-chromosome, which causes oocyte loss during childhood (Chapman et al., 2015; Kirshenbaum and Orvieto, 2019). Trisomy X, especially the 47XXX genotype, is associated with hypogonadotropic ovarian insufficiency (Dawood et al., 2018). Another congenital abnormality resulting in POI is the presence of CGG repeats (in the range of approximately 55–199 repeats) in the fragile-X mental retardation (*FMR1*) gene, which is also linked to Martin-Bell syndrome, which occurs in men (Allen et al., 2007; Oostra and Willemsen, 2009; Chapman et al., 2015). Alterations in the newborn ovary homeobox (NOBOX) and the factor in germline alpha (FIGLA), both oocyte-specific transcription factors, are involved in POI. NOBOX gene mutations cause oocyte loss after birth, while FIGLA mutations impair the regulation of zona pellucida genes, thus causing postnatal loss in primordial follicles (Zhao et al., 2008). Other mutations associated with POI are those occurring in forkhead box L2 (FOXL2) (Park et al., 2014), WT1 (Wilms tumor 1), NR5A1 (nuclear receptor subfamily 5 group A member1) (Lourenço et al., 2009), the transcription factors affecting folliculogenesis, are associated with POI. Abnormalities in bone morphogenetic protein 15 (BMP15) (di Pasquale et al., 2004), growth differentiation factor 9 (GDF-9), transforming growth factor-β superfamily, is also critical for POI (Rajkovic et al., 2004; Woad et al., 2006; Lourenço et al., 2009; Jiao et al., 2017; Qin et al., 2017). Mutations of the FSH receptor cause amenorrhea in the POI. It is also associated with FSH resistance hence raise serum FSH.

**TABLE 2:** Causative genes in POI.

Gene	Mutation rate (%)	Functional category	Regulatory mechanism	Reference
LHX8	N.A.	Transcription factor	Germ-cell-specific critical regulator of early oogenesis	Rossetti et al., 2017
SOHLH1*	N.A.	Transcription factor	Early folliculogenesis	Zhao et al., 2015
FOXO3A	2.2	Transcription factor	Regulating primordial follicle growth activation	Watkins et al., 2006; Gallardo et al., 2008
NOBOX(7q35)	1.0–8.0	Transcription factor	Follicle development	Rajkovic et al., 2004
FMR1(Xq27)	0.5–6.7	highly polymorphic CGG repeat in the 5′ untranslated region (UTR) of the exon 1	Transcriptional regulation	Oostra and Willemsen, 2009
PGRMC1(Xq22-q24)	0.5–1.5	Heme-binding protein	Regulation of apoptosis	Venturella et al., 2019
POLR3H	1.5	RNA polymerase III subunit H	Regulation of cell cycle, cell growth, and differentiation	Franca et al., 2019
GDF9(5q31.1)	0.5–4.7	Growth factor	Growth and differentiation of granulosa cell proliferation	Patiño et al., 2017b
BMP15(Xp11.2)	1.0–10.5	Growth factor	Growth and differentiation of granulosa cells (GCs)	di Pasquale et al., 2004
BMPR2	N.A.	BMP receptor	Signal transduction between oocytes and somatic cells	Patiño et al., 2017a
AMH(19p13.3)	2.0	Anti-Müllerian hormone	Control of the formation of primary follicles by inhibiting excessive follicular recruitment by FSH	Alvaro Mercadal et al., 2015
AMHR2(12q13)	1.0–2.4	AMH receptor	AMH signal transduction	Yoon et al., 2013
FOXL2(3q23)	1.0–2.9	Transcription factor	Differentiation and growth of granulosa cells	Bouilly et al., 2016
WT1(11p13)	0.5	Transcription factor	Granulosa cell differentiation and oocyte–granulosa cell interaction	Gao et al., 2014
NR5A1(9q33)	0.3–2.3	Transcription factor	Steroidogenesis in ovaries	Jiao et al., 2017
FSHR (2p21-p16)	0.1–42.3	Receptor	Follicular development and ovarian steroidogenesis	Welt, 2008
KHDRBS1	N.A.	Signal transduction activator	Alter mRNA expression level and alternative splicing	Wang et al., 2017
FIGLA (2p13.3)	0.5–2.0	bHLH transcription factor	Regulation of multiple oocyte-specific genes, including genes involved in folliculogenesis and those that encode the zona pellucida	Zhao et al., 2008
INHA variants	0–11	Growth factor	Maturation of ovarian follicles by FSH inhibition	Dixit et al., 2004
ESR1	N.A.	Estrogen receptor	Regulation of follicle growth and maturation and oocyte release	de Mattos et al., 2014
LHR	N.A.	Lutropin-choriogonadotropic hormone receptor	Regulation of ovarian follicle maturation, steroidogenesis, and ovulation	Simpson, 2008

*Identified from GWAS.

*N.A., not available.*

The steroidogenic factor 1 gene (*SF-1*, *NR5A1*) is important for gonadal differentiation and controls steroidogenesis by regulating steroidogenic acute regulatory protein (*StAR*) (Jehaimi et al., 2010), cytochrome P450, family 19, subfamily A, polypeptide 1 (*CYP19A1*) (Kim et al., 2011), lutropin-choriogonadotropic hormone receptor (*LHR*) (Simpson, 2008), and inhibin alpha subunit (*INHA*) genes (Dixit et al., 2004), which function in the hypothalamic–pituitary–steroidogenesis axis (Chapman et al., 2015). Recent discoveries have shown a link between POI development and spermatogenesis and oogenesis-specific basic helix-loop-helix (SOHLH) 1 and 2 sequence-specific DNA-binding factors (Dixit et al., 2004; Watkins et al., 2006; Gallardo et al., 2008; Zhao et al., 2015; Patiño et al., 2017b). These mutations cause infertility and loss of follicles.

#### Genome-Wide Association Study in POI

Genome-wide association study (GWAS), also known as whole-genome association study (WGAS), in POI involves genome-wide approaches to search for susceptible loci or genes that cause the disease (Qin et al., 2015b). GWAS is applied to investigate common genetic variants in different individuals to understand the association of a genetic component with POI in similar ethnicities. Owing to limited sample sizes and POI being rarer than PCOS or endometriosis, it has been difficult to identify plausible genetic candidates for POI (Jiao et al., 2018). Recent approaches using whole-genome sequencing and next-generation sequencing (NGS) have led to the identification of some causative genes from large POI pedigrees or shared genetic factors between POI and either natural age menopause or early menopause (Qin et al., 2015b). These causal genes from GWAS are marked with asterisks in Tables 2, 3 and are as follows: known POI candidates (SOHLH1 (Zhao et al., 2015), FSHR (Aittomäki et al., 1995; Huang et al., 2019); DNA damage repair-, homologous recombination-, and meiosis-related [stromal antigen 3 (STAG3)] (Heddar et al., 2019), synaptonemal complex central element protein 1 (SYCE1) (de Vries et al., 2014), scaffolding protein involved in DNA repair (SPIDR) (Smirin-Yosef et al., 2017), PSMC3 interacting protein (PSMC3IP) (Zangen et al., 2011), ATP-dependent DNA helicase homolog (HFM1) (Zhe et al., 2019), MutS protein homolog 4 and 5 (MSH4 and MSH5) (Carlosama et al., 2017; Guo et al., 2017; Wang et al., 2020), minichromosome maintenance component 8 and 9 (MCM8 and MCM9) (Wood-Trageser et al., 2014; AlAsiri et al., 2015; Desai et al., 2017), fusion protein (CSB-PGBD3) (Qin et al., 2015a), and nucleoporin 107 kDa (NUP107) (Weinberg-Shukron et al., 2015); and mRNA transcription- and translation-related [eukaryotic translation initiation factor 4E nuclear import factor 1 (eIF4ENIF1) (Zhao et al., 2019), and KH Domain-Containing, RNA-Binding, Signal Transduction-Associated Protein 1 (KHDRBS)] (Jiao et al., 2018). These data will allow further elucidation of the genetic mechanism underlying POI.

**TABLE 3:** Common POI-related genes in humans and mice.

Genes	Full name	Function	References
ATM	Ataxia telangiectasia mutated	A member of the phosphatidylinositol-3 kinase-like protein kinase (PIKK) family	Liu H. et al., 2020
BMP15	Bone morphogenetic protein 15	Growth factor beta	di Pasquale et al., 2004
BMPR1A/1B	Bone morphogenetic protein receptor, type IB	Growth factor beta	Renault et al., 2020
CLPP	ClPP caseinolytic peptidase, ATP-dependent, proteolytic subunit homolog (Escherichia *coli*)	Cytochrome P450 family 19 subfamily A member 1	Jenkinson et al., 2013
CSB-PGBD3*	CSB-PGBD3 fusion protein	DNA damage repair	Qin et al., 2015a
CYP19A1	Cytochrome P450, family 19, subfamily A, polypeptide 1	Cytochrome P450 family 19 subfamily A member	Kim et al., 2011
eIF4ENIF1*	Eukaryotic translation initiation factor 4E nuclear import factor 1	Regulates translation and stability of mRNAs in processing bodies	Zhao et al., 2019
ERCC6	DNA excision repair protein ERCC-6	Essential factor involved in transcription-coupled nucleotide excision repair	Qin et al., 2015b
FANCA	Fanconi anemia, complementation group A	DNA repair protein	Pyun et al., 2014
FOXL2	Forkhead box L2	Transcription factor	Park et al., 2014
FSHR	Follicle stimulating hormone receptor	Receptor	Aittomäki et al., 1995; Huang et al., 2019
HFM1*	HFM1, ATP-dependent DNA helicase homolog (*S*accharomyces *cerevisiae*)	Receptor	Zhe et al., 2019
MCM8/9*	Minichromosome maintenance component 8	DNA-repair gene	Wood-Trageser et al., 2014; AlAsiri et al., 2015; Desai et al., 2017
MSH4/5*	MutS protein homolog 4/5	Homologous recombination (HR) repair for DNA double strand breaks	Carlosama et al., 2017; Guo et al., 2017; Wang et al., 2020
NANOS3	Nanos homolog 3 (Drosophila)	Signaling molecule	Wu et al., 2013
NBN	Nibrin	DAN-repair gene	Chrzanowska et al., 2010; Tucker et al., 2018
NR5A1	Nuclear receptor subfamily 5, group A, member 1	Receptor	Lourenço et al., 2009
NUP107*	Nucleoporin 107 kDa	Receptor	Weinberg-Shukron et al., 2015
PGRMC1	Progesterone receptor membrane component 1	Cell cycle gene meiotic recombination	Peluso, 2013
PRIM1	DNA primase small subunit	DNA replication	Stolk et al., 2012
PSMC3IP*	PSMC3 interacting protein	Cell cycle genes	Zangen et al., 2011
SALL4	Spalt-like transcription factor 4	Oogenesis	Wang et al., 2019
SGO2	Shugoshin 2	Transcription factors	Faridi et al., 2017
SOHLH1	Spermatogenesis and oogenesis specific basic helix-loop-helix 1	Cell cycle genes	Zhao et al., 2015
SPIDR*	Scaffolding protein involved in DNA repair	Homologous recombination repair during meiosis	Smirin-Yosef et al., 2017
STAG3*	Stromal antigen 3	DNA-damage	Heddar et al., 2019
StAR	Steroidogenic acute regulatory protein	Acute regulation of steroid hormone synthesis	Jehaimi et al., 2010
SYCE1*	Synaptonemal complex central element protein 1	Growth factor beta	de Vries et al., 2014
WRN	Werner syndrome protein; Werner syndrome, RecQ helicase-like	Caseinolytic mitochondrial matrix peptidase	Du et al., 2004

*Identified from GWAS.

#### Non-coding RNA in POI

The role of non-coding RNAs (ncRNAs) in biology has become an area of intense focus, since they have already been extensively studied for the determination of altered protein function in various diseases (Carninci et al., 2005; Elgar and Vavouri, 2008; Djebali et al., 2012). RNAs that do not encode conventional proteins are collectively referred to as ncRNAs, which function as epigenetic regulators. MicroRNAs (miRNAs) are endogenously present in mammalian ovaries (Ahn et al., 2010) and their expression patterns are altered throughout ovarian development and folliculogenesis (Veiga-Lopez et al., 2013), implying their functional roles in the ovarian cycle. The list of miRNA-related POI studies is presented in Table 4.

**TABLE 4:** List of miRNA-related POI studies.

miRNAs	Association with POI	Reference
miR-146 miR-196a2	Putative gene-gene interaction between miR-146 and miR-196a2 may be involved in POF development of Korean women.	Rah et al., 2013
	MiR-146aC > G and miR-196a2T > C change the mRNA expression patterns in granulosa cells.	Cho et al., 2017
miR-146	The expression of miR-146a in plasma and in ovarian granulosa cells of patients with POI was significantly upregulated.	Chen et al., 2015
miR-23a	Mir-23a may play important roles in regulating apoptosis via decreasing XIAP expression in human ovarian granulosa cells of POI patients.	Yang et al., 2012
miR-22-3p	The decreased expression of miR-22-3p in plasma of POI patients may reflect the diminished ovarian reserve and be a consequence of the pathologic process of POI.	Dang Y. et al., 2015
miR-379-5p	MiR-379-5p, PARP1, and XRCC6 were differentially expressed in granulosa cells of biochemical POI.	Dang et al., 2018
miR-21	Low expressions of miR-21 and Peli1 were detected in autoimmune POI mice and patients.	Li et al., 2020
miR-127-5p	The upregulation of miR-127-5p was also detected in plasma of bPOI (biochemical POI) individuals.	Zhang et al., 2020

Recently, the mechanism of lncRNA HCP5 was reported to be responsible for human POI, that is, HCP5 regulated MSH5 expression and granulosa cell function by directly binding with YB1 and modulating its subcellular localization. This study also discovered a novel lncRNA HCP5 that contributes to dysfunctional granulosa cells by transcriptionally regulating MSH5 and DNA damage repair via YB1, providing a novel epigenetic mechanism for POI pathogenesis (Wang et al., 2020). Multiple studies have reported that downregulation of nc-RNAs has also been found in POI women, which may lead to new clinical markers to identify POI, as well as potential therapeutics for POI. Nc-RNA has provided a novel foundation for the discovery of markers for specific diseases, and therapeutics have developed pipelines for treating POI. An improved understanding of ncRNA biology and the development of a delivery system will contribute to the identification and treatment of multiple diseases in patients.

### Non-genetic Causes of Premature Ovarian Insufficiency: Autoimmune, Iatrogenic, Metabolic, Infectious, or Environmental Factors

Autoimmunity is responsible for 4–30% of the cases of POI (Silva et al., 2014; Ebrahimi and Akbari Asbagh, 2015). Systemic pro-inflammatory conditions have a negative impact on follicular dynamics, leading to an alteration of ovarian homeostasis (Kirshenbaum and Orvieto, 2019). Although the main targets for attack by autoimmunity are the steroid-producing cells in the pre-ovulatory follicles and corpora lutea, sometimes follicular depletion, fibrosis, and abnormal activation of epithelial cells are observed (Warren et al., 2014; Sullivan et al., 2016; Kirshenbaum and Orvieto, 2019). The occurrence of other autoimmune disorders, such as lymphocytic oophoritis and the presence of anti-ovary antibodies, is an indication of autoimmune etiology in POI (Bakalov et al., 2005; Fenton, 2015; Kirshenbaum and Orvieto, 2019). Lymphocytic oophoritis is more commonly seen in Addison’s disease and adrenal immunity-associated POI than in isolated POI (Hoek et al., 1997). Evidence of lymphocytic oophoritis is histopathologically found in ovarian biopsies of women with normal karyotypes experiencing amenorrhea (Falorni et al., 2014). Hypothyroidism, autoimmune adrenal insufficiency, autoimmune polyglandular syndrome, and autoimmune Addison’s disease are the most common autoimmune diseases associated with POI. Pelvic tuberculosis has been found to cause POI in 3% of patients. In addition, smoking can generate POI through polycyclic hydrocarbons in smoke. The most frequent iatrogenic causes of POI are both chemotherapy and radiotherapy. whose effects on the ovary are highly variable depending on gonad toxicity (Fenton, 2015). Metabolic causes of POI include galactosemia, myotonic dystrophy, and hydroxylase deficiency. Lastly, prolonged use of gonadotropin-releasing hormone (GnRH) therapy may also lead to ovarian suppression.

## Animal Models for Understanding POI

Mouse and rat models are used to identify the genetic and molecular mechanisms involved in POI as well as to gain insights for the development of novel therapeutics. Both animals have a high degree of similarity with humans with respect to ovarian developmental processes and functions, and exhibit a similar genetic pathway regulation responsible for POI, except for some variations and physical differences, including ovulation time and mono versus polyovulation (Na and Kim, 2020). The most common animal models of POI have been generated by chemical induction with cyclophosphamide, busulfan, cisplatin, and murine ZP3 330–342 peptides (ZP3) (Table 5), which can be administered via intraperitoneal (i.p.) or tail-vein (intravenous, i.v.) injection (Na and Kim, 2020). Ovarian failure can be confirmed using vaginal smears (Na and Kim, 2020).

**TABLE 5:** Chemical and peptide-induced animal model of POI.

Chemical, dose, and duration	Reference
Cyclophosphamide (120 mg/kg), a single i.p. injection for 2 weeks*	Nguyen et al., 2019; Ding et al., 2020a
Busulfan (20 mg/kg) or (12 mg/kg) and cyclophosphamide (70 mg/kg) or (200 mg/kg), a single i.p. injection for 2 weeks*	Mohamed et al., 2018; Yang et al., 2019
Murine ZP3 330–342 peptides (NSSSSQFQIHGPR) (1 mg/mL), injection twice every 2 weeks* (autoimmune POI model)	Li et al., 2020
cisplatin (5 mg/kg), a single i.p. injection for 2 weeks*	Nguyen et al., 2019

*Dose and time of injection vary between studies.

A naturally aged model (NOA) was also used to study the mechanisms of the premature termination of ovarian function. Mice or rats aged 12–14 months showed an ovarian failure and decreased follicles (Li et al., 2017). Ovariectomized (OVX) models have been employed as well, as ovarian surgical injury causes a premature termination of ovarian function (Erler et al., 2017).

Some of the results of the genetic studies on POI in mice were later confirmed in human cases of POI, including those related to NOBOX (Rajkovic et al., 2004; Qin et al., 2007; Jiao et al., 2017; Table 3). In addition to the numerous genes that have been already demonstrated to be linked to human POI, other target genes have been discovered after investigations in animal models, including follitropin receptor KO (FORKO) (POI developmental model) and Bax KO (ovarian function enhancer model) mice (Na and Kim, 2020). Most of the genes related to POI in mice have shown a particular phenotypic change. To date, many of the gene mutations reported to cause POI in humans are common with those determined in the mouse models of POI. A list of candidate genes and their observed phenotypes in the mouse models are included in Table 4.

In POI etiology, complex inheritance may also have a critical role, but this remains difficult to prove. To date, all known genetic causes of POI have been monogenic and Mendelian. Although NGS has accelerated the identification of the genes involved, the complex inheritance of POI and the possible genetic or environmental factors remain to be explored. A complex inheritance with a proper gene regulation network may be involved in POI signaling and studying a large amount of sequencing data of POI patients could help explore this possibility. Several studies have also reported ovarian failure induced by exogenous treatments, including radiation and chemotherapy. These studies showed that POI is fully rescued in mice by the transplantation of human mesenchymal stem cells derived from menstrual blood, cord blood, or exosomes isolated from different human tissues (Dang J. et al., 2015; Lai et al., 2015). Other potential treatments for POI, which were first applied to mice, are Hippo pathway disruption by mechanical ovarian fragmentation and AKT pathway activation by chemical drug administration. After showing promise in mouse models, the therapy was also attempted in POI patients, resulting in a live birth (Kawamura et al., 2013). With advances in NGS and gene editing techniques, the generation of mouse models has been made easier, promoting the study of potential therapies for genetics-based POI. Therapies may be designed for specific mutations or specific gene defects.

## Small Extracellular Vesicles May Allow Recovery From POI

Small extracellular vesicles (sEVs) derived from stem cells play a role in tissue regeneration and hence, are postulated to have therapeutic value for damaged ovaries (Igboeli et al., 2020; Liu M. et al., 2020; Zhang et al., 2021). Stem cells are therapeutic because of their ability to multiply indefinitely and differentiate into different types of cell lineages.

Small extracellular vesicles have been shown to function in POI, triggering several signaling pathways including PI3K/AKT, sirtuin (SIRT) 4, and SIRT7 (Ding et al., 2020a,b; Liu M. et al., 2020; Yang et al., 2020). The PI3K/AKT pathway is important in the activation of primordial follicles, which are important for folliculogenesis as well as proliferation of granulosa cells by regulating apoptosis (Chen et al., 2020). Embryonic stem cell-derived sEVs (ESC-sEVs) have been shown to restore E2, FSH, and AMH, increase the number of normal follicles, and decrease the number of atretic follicles upon transplantation in a CTX and BUS-induced POI mouse model, which functioned to recover ovarian function (Liu M. et al., 2020). ESC-sEVs lead to reduction in the expression of apoptosis-related proteins, such as Bax and caspase-3 and increase in the expression of Bcl-2 (da Silveira et al., 2017). Yang et al. (2020) also demonstrated that human umbilical cord mesenchymal stem cell-derived (HucMSC)-sEVs stimulate primordial follicles by activating the oocyte PI3K/mTOR pathway by carrying functional miRNAs such as miR-146a-5p or miR-21-5p. Activation of the oocyte PI3K/mTOR signaling pathway results in stimulation of primordial follicles and acceleration of follicular development after kidney capsule transplantation. Intrabursal injection of HucMSC-exosomes in aged female mice was shown to promote recovery from defects in fertility with increased oocyte production and improved oocyte quality. A recent study suggested that miR-17-5p from HucMSC-sEVs improves ovarian function in POI by regulating SIRT7 (Ding et al., 2020b). The numbers of antral and total follicles increased significantly in the ovaries of the exosome-treated CTX-POI mice. HucMSC-derived exosomal miR-320a regulates ADP/ATP translocase 2 (ANT2), AMP-dependent kinase (AMPK), and long form of OPA1 (L-OPA1) via SIRT4, which prevents reactive oxygen species production (Ding et al., 2020a). Introduction of anti-miR-320a in a CTX-induced POI mouse model led to downregulation of SIRT4 target genes. Overall, sEVs from stem cells have been suggested as signaling regulators in POI, which could be a therapeutic strategy against the disease.

## Conclusion

There has been a gradual increase in the occurrence of POI, which is highly heterogeneous with known causative genes and influences a variety of biological activities, including hormonal signaling, metabolism, development, DNA replication, DNA repair, and immune function. Only a small percentage of patients can be accounted for by the presently known POI genes; a larger number of the patients remain without a genetic diagnosis. Recognizing the genetic origin of POI can pose a major advantage for patients and their families. Screening of family members can be achieved when the genetic cause has been pinpointed, thus facilitating timely intercession with HRT to reduce the requirement for the cryopreservation of ova and to increase future fertility potential. Improving prognosis and fertility potential is possible when there is a better understanding of the genetic causes of POI. This understanding would also facilitate better patient guidance and management. Crucial insights for the development of therapies necessary for ovarian function may be obtained by discerning new genes and pathways that play vital roles in POI development, and may lead to new drug therapies. The promotion of widespread awareness should be upheld to inform women with POI on reducing the risk factors for cardiac illnesses by avoiding smoking, having regular routine exercise, and having a healthy weight. Annual monitoring of patients should be carried out, especially in terms of smoking history, healthy weight, and blood pressure abnormalities, which should serve as the key cardiovascular assessment parameters. The centerpiece of POI treatment is appropriate counseling, psychological support, and HRT, which should be recommended for all women with POI. Additionally, promising methods for screening for POF are currently being developed.

## Author Contributions

SC and M-SY designed the manuscript. ZU, SC, and M-SY wrote the original draft of the manuscript, reviewed, and edited the manuscript. All authors read and approved the final manuscript.

## Conflict of Interest

The authors declare that the research was conducted in the absence of any commercial or financial relationships that could be construed as a potential conflict of interest.
